# The Effects of *Tremella fuciformis* Polysaccharide on the Physicochemical, Multiscale Structure and Digestive Properties of *Cyperus esculentus* Starch

**DOI:** 10.3390/foods13091425

**Published:** 2024-05-06

**Authors:** Shanshan Zhang, Yingxu Liu, Tong Sun, Hongcheng Liu, Dawei Wang

**Affiliations:** 1 School of Food Science and Engineering, Jilin Agricultural University, Changchun 130118, China; winkyshanshan@163.com (S.Z.); liuyingxu2023@163.com (Y.L.); suntong08051320@163.com (T.S.); liuhongcheng@jlau.edu.cn (H.L.); 2 Engineering Research Center of Grain Deep-Processing and High-Effeciency Utilization of Jilin Province, Changchun 130118, China; 3 Scientific Research Base of Edible Mushroom Processing Technology Integration of Ministry of Agriculture and Rural Affairs, Changchun 130118, China; 4 Key Laboratory of Technological Innovations for Grain Deep-Processing and High-Effeciency Utilization of By-Products of Jilin Province, Changchun 130118, China

**Keywords:** *Cyperus esculentus* starch, digestive properties, interrelation, multiscale structure, *Tremella fuciformis* polysaccharide

## Abstract

In this study, we have investigated the effects of *Tremella fuciformis* polysaccharide (TP) on the pasting, rheological, structural and in vitro digestive properties of *Cyperus esculentus* starch (CS). The results showed that the addition of TP significantly changed the pasting characteristics of CS, increased the pasting temperature and pasting viscosity, inhibited pasting, reduced the exudation of straight-chain starch and was positively correlated with the amount of TP added. The addition of the appropriate amount of TP could increase its apparent viscosity and enhance its viscoelasticity. The composite system of CS/TP exhibited higher short-range ordered structure and solid dense structure, which protected the crystal structure of CS, but was related to the amount of TP added. In addition, the introduction of TP not only decreased the in vitro digestion rate of CS and increased the content of slow-digestible starch (SDS) and resistant starch (RS), but also reduced the degree of digestion. Correlation studies established that TP could improve the viscoelasticity, relative crystallinity and short-range order of the CS/TP composite gel, maintain the integrity of the starch granule and crystalline structure, reduce the degree of starch pasting and strengthen the gel network structure of CS, which could help to lower the digestibility of CS.

## 1. Introduction

**Figure 1:**
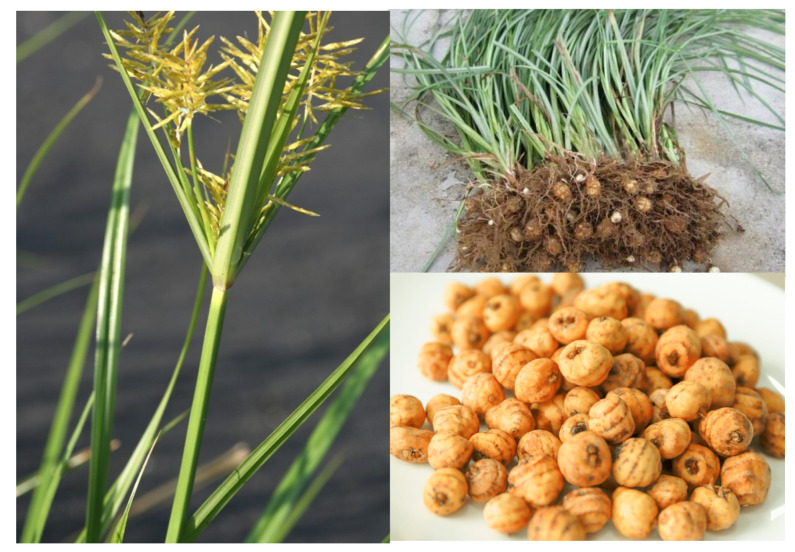
*Cyperus esculentus*.

*Cyperus esculentus*, also known as tiger nut, oil saxifrage, underground chestnut and ginseng bean (Figure 1), belongs to the genus Saxifrage of the family Saxifrage, annual herbaceous plants, and is widely distributed in Europe, South America, Africa and Asia (including China) [1]. It was officially listed as a new type of health food in China in 2022, and since its introduction to China, it has been widely planted mainly in the northern part of the country [2]. *Cyperus esculentus* is rich in nutrients, mainly containing 26–38% fat, 25–30% starch, 15–20% sugar and 3–15% protein, making it a grain and oil crop of high comprehensive use value [3,4]. Previous studies of *Cyperus esculentus* have focused on the extraction and quality enhancement of *Cyperus esculentus* oil due to the plant’s high oil content. *Cyperus esculentus* oil is appreciated for its richness in unsaturated fatty acids (>60%) and trace minerals, bright colour and nutty aroma, and is comparable to olive and hazelnut oils [5,6]. In addition, *Cyperus esculentus* starch (CS) is also a major component of *Cyperus esculentus* and a major by-product of oil extraction. The deep processing of CS can increase its added value and promote the comprehensive development and utilisation of *Cyperus esculentus* resources. CS is an odourless white powder in the form of ellipsoidal or spherical granules with diameters ranging from 2 to 17 μm [7]. Relevant studies have shown that CS is a type A starch, with a ratio of straight-chain to branched-chain starch of 1:3. It has good transparency, solubility and swelling, and its gel-forming ability, freeze-thaw stability and ageing characteristics are better than those of potato starch and sweet potato starch, so it is a high-quality starch raw material with a better prospect for development [8,9,10]. In view of these properties, it is mainly used in jellies, cold beverages, and confectionery as a thickening gel [4]. Recently, researchers have paid more attention to the effects of some non-starch polysaccharides on the starch pasting and rheological properties of CS. It was found that different additions of CQSG could improve the rheological and pasting characteristics of CS [11]. In a study by Miao et al., it was found that the structural and functional properties of CS could be further modified by using dry heat and CQSG synergistically to enhance its freeze–thaw and thermal stability [12].

**Figure 2:**
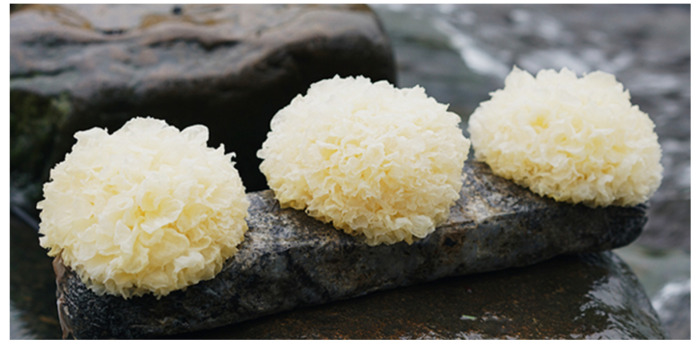
*Tremella fuciformis*.

In recent years, edible mushroom polysaccharides have been shown to play a role in improving insulin resistance and inhibiting starch-digesting enzymes, in addition to possessing properties such as thickening, stabilising and gelation of non-starch polysaccharides [13]. They have been widely used to improve the pasting, rheological and digestive properties of starch [14]. *Tremella fuciformis* is a type of nourishing edible fungus widely cultivated in China, which contains polysaccharides, proteins, amino acids and a variety of trace elements, among which polysaccharides are the main active components (Figure 2). *Tremella fuciformis* polysaccharide (TP), as one of the important active components of *Tremella fuciformis*, possesses a wide range of processing properties, such as thickening, gelling and emulsification, which are mainly attributed to its complex structure. The structure of the linear backbone of TP consists of α-1,3-linked D-mannan residues, and the side chain is composed of α-1,3-linked D-glycan residues, while the side chains usually contain glucosyl, mannosyl, focusyl, xylosyl and glucuronide substituents, which are usually attached to the main chain [15,16,17]. In addition, TP shows a variety of physiological activities, such as antioxidant, immunity promotion, maintenance of colonic bacteria, stabilisation and assistance in lowering blood lipids [18,19,20]. The incorporation of TP into starch-based foods can utilise its functions in inhibiting starch digestion and regulating the postprandial glycaemic effect [21,22]. Therefore, it is of interest to investigate the use of edible mushroom polysaccharides to improve the processing characteristics of starch such as pasting, regeneration and rheology while slowing down the rate of starch digestion.

However, existing studies on the effect of TP on processing characteristics such as pasting, rheology and digestive properties of CS are not clear. Therefore, this study investigates the effect of different additions of TP on the pasting, thermal, rheological and in vitro digestive properties of CS. It focuses on the interaction between TP and CS molecules during pasting, rheology and digestion, and also analyses the correlation between pasting, structural and in vitro digestive property parameters, which will provide useful information for the development of low-digestible and healthy CS–TP starch-based food products in the promotion of product upgrading in the CS industry.

## 2. Materials and Methods

### 2.1. Materials

*Tremella fuciformis* was purchased from local supermarkets. TP was extracted and purified according to the method of Chen et al. [23]. Its purity was about 85.5%. *Cyperus esculentus* were provided by Jilin Province tiger nuts oils Agricultural Technology Co. (Chuangchun, China) Amyloglucosidase (100,000 U/g) and porcine pancreatic α-amylase (50,000 U/g), glucose oxidase-peroxidase and straight-chain amylose assay kits were purchased from Solebo Bio-technology Co., Ltd. (Beijng, China). All other reagents were analytical reagents.

### 2.2. Preparation of CS

First, according to the pre-laboratory test [24], the method of supercritical CO_2_ was used to extract oil. The residue was crushed to 200 mesh, stirred continuously with 0.1 mol/L aqueous sodium hydroxide solution (solid-liquid ratio, 1:5, g/mL) for 6 h at room temperature, and then centrifuged at 3800 rpm for 20 min to extract the resulting precipitate, which was precipitated and resuspended four times in distilled water, and then the obtained precipitates were vacuum freeze-dried for 24 h. The starch was carefully milled and passed through a 200-mesh sieve, and finally collected as *Cyperus esculentus* starch (CS), a starch with a purity of 90.8% (approx.), containing approximately 6.24% moisture, 0.55% fat, 0.32% protein and 0.29% ash.

### 2.3. Preparation of CS/TP Starch Composite System

A mixture of TP (0, 0.2%, 0.4%, 0.8% and 1.0%, *w*/*v*) and CS (6%, *w*/*v*) (TP/CS) was prepared as follows. First, different masses of TP were dispersed in distilled water, vortexed for 2–3 min, magnetically stirred for 30 min, then heated in a thermostatic water bath at 60 °C for 20 min with continuous stirring, and finally cooled to room temperature. Next, a certain mass of CS was dispersed into the TP suspension according to the weighing and stirred continuously for 30 min at room temperature to obtain a homogeneous composite suspension, and finally the content of CS in the composite system was made to be 6% (*w*/*v*), and the samples were named CS, CS-TP0.2%, CS-TP0.4%, CS-TP0.6%, CS-TP0.8% and CS-TP1.0% (*w*/*v*).

### 2.4. Determination of Pasting Characteristics

The pasting characteristics of CS and CS/TP composite systems were determined using a rapid viscosity analyser (RVA-TecMaster) (RVA, TecMaster, Perten Instruments, Warriewood, Australia). The procedure was as follows: First, a certain mass of the sample was accurately weighed and placed into a special test cassette of RVA. Subsequently, the stirring programme was initiated for 60 s. Then, we followed the previous method [25], using the RVA procedure to test the adhesive properties of the samples. The specific test procedure was set as follows: the initial temperature was 50 °C and held for 1 min; then, the temperature was increased to 95 °C at a rate of 12 °C/min and held at 95 °C for 2.5 min; subsequently, the temperature was again reduced to 50 °C at a rate of 12 °C/min and, finally, held at 50 °C for 2 min. This series of temperature change processes allowed us to collect a series of pasting parameters, which included peak viscosity (PV), trough viscosity (TV), disintegration viscosity (BV), final viscosity (FV), regeneration viscosity (SV) and pasting temperature (PT). These parameters provide us with detailed information about the pasting characteristics of CS and CS/TP composite systems.

### 2.5. Measurement of Starch Leaching

The samples were prepared according to the pasting method described in Section 2.4. They were collected at the end of pasting and centrifuged at 8000× *g* for 40 min. The resulting supernatant was diluted as appropriate, and the amount of starch leachate during the pasting process was determined using an enzyme marker (Synergy H1, BioTek Instruments Co., Ltd., Winooski, VT, USA) at 620 nm [25]. Meanwhile, the standard curve for the content of straight-chain starch was determined according to the kit instructions: y = 0.0582x − 0.057 (R^2^ = 0.9988). The amount of straight-chain starch leached from the samples was calculated from the standard curve of starch. 

### 2.6. Determination of Swelling and Solubility

Referring to previous methods [11], with slight modifications, CS or CS/TP (0.2%, 0.4%, 0.6%, 0.8%, 1.0%) mixtures (300 mg) were accurately weighed and mixed well with 15 mL of distilled water in a centrifuge tube. Subsequently, these slurries were heated in a thermostatic shaker water bath at 50 °C, 60 °C, 70 °C, 80 °C and 95 °C for 1 h. After the mixtures were cooled to room temperature, they were centrifuged at 3000 rpm for 15 min. After centrifugation, the supernatant was carefully poured out and the remaining starch sediment was weighed. Next, the supernatant was transferred to a weighing flask and dried at 105 °C until constant weight. To ensure the accuracy of the experimental results, all experimental steps were repeated more than 3 times. The solubility (SOL) and swelling (SP) were calculated as follows:

$$SOL(\%)=\frac{A}{W}\times 100\%$$

$$SP(g/g)=\frac{P}{W(100-SOL)}$$

where A is the weight of the dried supernatant (g), W is the weight of the sample (g) and P is the weight of the precipitate (g).

### 2.7. Determination of Rheological Properties

#### 2.7.1. Determination of Static Rheological Properties

The CS/TP samples of each group were prepared as described in Section 2.4, and the rheological properties of the samples were measured by a rheometer (DHR-1, TA Instruments Ltd., New Castle, DE, USA), with a plate fixture of 40 mm in diameter and a gap set to 1000 μm. Following the method of Ning et al. [26], the apparent viscosity of the samples was recorded as a function of the shear rate at a temperature of 25 °C with the shear rate set from 0.01 s^−1^ to 500 s^−1^, and the power law equation was used to fit and analyse the curve of specific apparent viscosity as a function of shear rate.

$$\eta =K\times \gamma^{n-1}$$

where η is the apparent viscosity (Pa-s), γ is the shear rate (s^−1^), K is the coefficient of consistency (Pa-s)^n^ and n is the flow behaviour index.

#### 2.7.2. Determination of Dynamic Viscoelasticity

CS and CS/TP composite gels were prepared according to the pasting method described in Section 2.4, and the dynamic viscoelasticity of the starch pastes was determined using a rheometer (DHR-1, TA Instruments Ltd., New Castle, DE, USA). A 40 mm diameter flat plate fixture was selected, the gap was set at 1000 μm, the oscillatory strain was set at 1% and the temperature was set at 25 °C. The effects of frequency changes from 0.1 to 10 Hz on the storage modulus (G′) and loss modulus (G″) were observed.

### 2.8. In Vitro Digestion Characteristics and In Vitro First-Order Digestion Kinetics Fitting

Groups of CS and CS/TP samples were prepared according to the pasting method described in Section 2.4 and lyophilised to obtain groups of lyophilised sample powders. The in vitro digestibility of each group of samples was determined as described by the previous authors with slight modifications [26,27]. In brief, 200 mg of each sample was weighed into a beaker, and 10 mL of sodium acetate buffer solution (pH 5.2, 0.2 mo/L) was added and mixed thoroughly. The samples were pasted in a water bath with magnetic stirring (95 °C) for 30 min and transferred to a water bath shaker at 37 °C to equilibrate for 10 min. Then, 10 mL of newly configured enzyme mix solution (α-amylase 200 U/mL and amyloglucosidase 160 U/mL) was added to each sample and immediately placed in a 37 °C water bath with constant shaking for treatment at 0, 20, 40, 60, 90, 120 and 180 min time points, 0.5 mL of hydrolysate was removed and 1.5 mL of 95% ethanol solution was added to inactivate the enzyme treatment. Then, the supernatant was taken after centrifugation at 5000 rpm for 10 min, and the glucose content was analysed in the supernatant by glucose oxidase–peroxidase (GOPOD) kit Glucose content, and the glucose content was determined by an enzyme marker at 510 nm. The formulae for RDS, SDS and RS content were as follows:

$$RDS\left(\%\right)=\frac{\left(G_{20}-FG\right)\times 0.9}{TS}\times 100$$

$$SDS\left(\%\right)=\frac{\left(G_{120}-G_{20}\right)\times 0.9}{TS}\times 100$$

$$RS\left(\%\right)=(1-RDS-SDS)\times 100$$

where RDS, SDS and RS represent rapidly digested starch, slowly digested starch and resistant starch, respectively; FG, G20 and G120 are the amount of glucose released from the digestate after 0, 20 and 120 min of digestion, respectively; TS is the total starch mass; and 0.9 is the glucose-based conversion factor.

Meanwhile, the digestion kinetics of the samples were analysed to fit the starch digestion process using the first-order kinetic equation. The formulae was as follow:

$$C_{t}=C_{\infty }\times (1-e^{-kt})$$

where C_t_ denotes the amount of starch digested at the corresponding time t, C_∞_ is an estimate of the amount of starch digested at the end of digestion and *k* denotes the starch digestibility constant (min^−1^).

### 2.9. Measurement of Particle Size Distribution

Particle size distributions of CS and CS/TP composite systems were characterised using a laser scattering particle size analyser (Mastersizer 3000, Malvern Instruments Ltd., Malvern, UK) according to our previous methods [28]. The values of volume-averaged diameters D(4,3), D(3,2), D(10), D(25), D(50), D(75) and D(90) were recorded.

### 2.10. Determination of Crystallinity (XRD)

Lyophilised samples of CS and CS/TP complexes, mixed with unpasteurised and RVA-prepared CS and CS/TP complexes, were tested on an X-diffractometer (MiniFlx 600, Nihon Rikkyo Co., Ltd., Tokyo, Japan), with a set tube pressure of 40 kV and a set tube flow of 40 mA. The scanning range was 5–40°, the scanning rate was 4°/min and the scanning step was 0.02°. The relative crystallinity was calculated using MDI jade 6.0 software. Relative crystallinity was calculated using MDI jade 6.0 software [29].

### 2.11. Short-Range Ordered Measurements (FT-IR)

Groups of CS and CS/TP samples were prepared according to the pasting method described in Section 2.4 and lyophilised to obtain groups of lyophilised sample powders. The infrared spectral information of each group of samples was determined according to an infrared spectrometer (Nicolet iS20 spectrometer, Thermo Fisher Scientific, Waltham, MA, USA) [30]. The samples (2 mg) were ground and mixed with potassium bromide (200 mg) and then pressed and measured. The number of scans was 32, and the number of scanned waves ranged from 400 to 4000 cm^−1^, with a resolution of 4 cm^−1^. The baseline was calibrated and deconvolution processed using OMNIC 9.2 software for (800–1200 cm^−1^).

### 2.12. Cryo-Scanning Electron Microscopy (Cryo-SEM)

Groups of CS and CS/TP samples were prepared according to the pasting method described in Section 2.4 and stored at 4 °C for 24 h to form a gel. A small number of samples was placed on a pegged sample stage and pre-cooled at −25 °C, so that the samples were rapidly frozen to a glassy state, and the microstructures of each group of samples were observed under a cryo-electron microscope (Phenom Pro, Feiner Science Ltd., Eindhoven, The Netherlands). Experimental conditions: voltage value—5 kV; magnification 5000×.

### 2.13. Statistical Analysis

All experimental data analyses were conducted in three parallel trials and the test result data are represented as mean ± standard deviation. Origin 2022 was used for graphing and SPSS 2022 was used for one-way ANOVA and Duncan’s method of multiple range comparisons of the resultant data of the parallel test groups. *p* < 0.05 indicated a significant difference. The correlation between the extradigestive properties of the CS/TP composite system and the degree of pasting, swelling power, starch leaching, crystallinity and molecular ordering was assessed using Pearson’s test.

## 3. Results

### 3.1. Paste Characterisation

The pasting parameters of CS/TP complexes are shown in Table 1. TP significantly altered the pasting parameters of CS, such as increasing the pasting temperature and viscosity (peak viscosity, trough viscosity and final viscosity), and the effect was related to the amount of TP added. When the amount of TP was increased, the pasting viscosity was significantly higher (*p* < 0.05) compared to the control CS. This may be due to the high water absorption of TP resulting in the reduction of water available in the CS/TP blend system and the weakening of hydration in the amorphous region of the starch granules, which made the CS/TP blend system more difficult to paste and the pasting temperature higher. This is in line with previous studies of *Mesona chinensis* polysaccharide; AAP increases the viscosity of starch throughout the pasting stage of the process [31,32]. The reason for this finding could also be that the polysaccharides interacted with the swollen starch and its leached starch fractions (mainly straight-chain starch), which on the one hand promoted the formation of a tighter and more complex three-dimensional network structure, and the conjugates subsequently attached to the surface of starch granules, which suppressed further swelling of the granules and the leakage of starch fractions, resulting in an increase in the pasting temperature of starch, and the increase in peak viscosity of starch. The peak viscosity of starch increased and even caused incomplete starch pasting [33,34]. In addition, the increase in the viscosity value of starch pasting after the addition of polysaccharides may be due to the interaction of hydrophilic polymers with dissolved low molecular weight branched-chain starch and straight-chain starch molecules [35].

The disintegration value is the difference between peak viscosity and trough viscosity, which indicates the degree of damage and stability of starch granules. It is mainly used to indicate the stability capacity and damage degree of starch granules under the heat process; the larger the disintegration value, the poorer the thermal stability of the starch granules [36], as can be seen from Table 1. The disintegration value showed a tendency to decrease and then increase with the increase of TP concentration, and the disintegration of the complex was significantly lower than that of CS when the polysaccharide concentration was 0.6%, which indicated that the moderate addition of TP could improve the resistance of the CS to heat treatment and machinery. It is likely that a certain amount of polysaccharide can be wrapped around the surface of starch granules during the pasting process, resulting in the formation of tighter cross-links between the polysaccharide and the starch granules, which in turn inhibits the swelling and fragmentation of the starch granules. In addition, the reduced thermal stability of CS/TP complexes with a high added amount of TP was mainly due to the fact that high-temperature shear weakened the thickening ability of glucuronoxylomannan in TP during the pasting process, which reduced the thermal stability of starch [37].

The rise value is the difference between the final viscosity and the valley viscosity on the RVA pasting curve, reflecting the extent to which the viscosity of the starch paste rises during the cooling process [38]. As shown in Table 1, the rapid increase in viscosity during cooling of CS paste indicates that CS is prone to short-term regrowth, and the starch molecular chains are aggregated together through hydrogen bonding to form a gelatinous mesh structure. In contrast, the addition of a small amount of TP (0.2%) was effective in reducing the final viscosity and the rebound value. The decrease in the regain value also suggests that TP may interact with starch molecules, and this interaction will to some extent reduce the interactions between straight-chain starch molecules. All other CS/TP complexes showed higher rebound values than CS, which may be due to the partial incompatibility of dissolved polysaccharides and starch in the continuous phase. Therefore, the selection of a suitable concentration of TP is important to control CS pasting or ageing behaviour.

### 3.2. Determination of Leached Amount of Straight-Chain Starch

**Figure 3:**
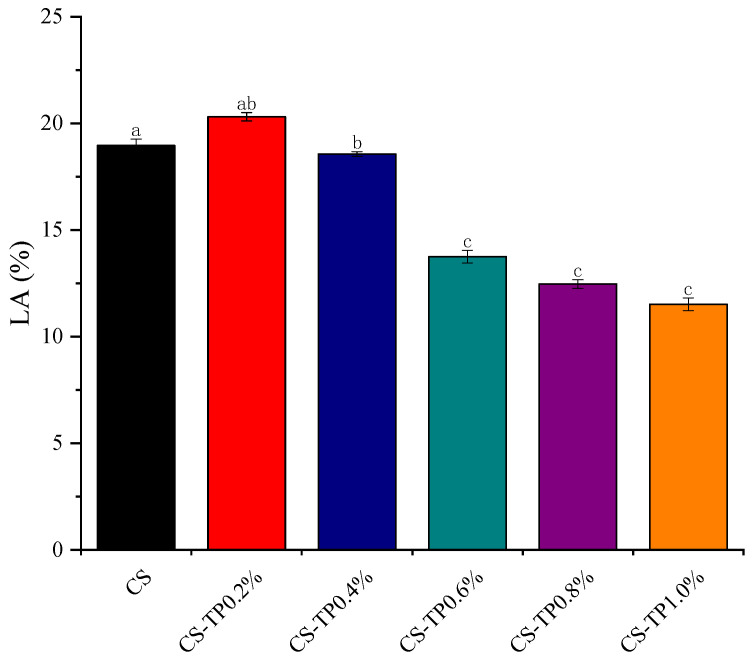
Leaching amylose of CS/TP composite systems. a–c: Different lowercase letters indicate significant difference (*p* < 0.05).

Amylose has an important role in starch pasting and regeneration, so we investigated the amount of amylose leakage (LA) during the pasting process of the composite system. When the starch granules were gelatinised, the starch granules were broken and the amylose was leached out and dissolved. The effect of different additions of TP on the amount of soluble starch leakage during the CS pasting process is shown in Figure 3. With the increase of TP addition, the difference in amylose leakage between different complexes appeared, and the trend of amylose leakage appeared to increase first and then decrease. This may be due to the fact that a small amount of TP may be bound on the surface of CS granules through hydrogen bonding and in other ways, similar to connecting hydrophilic chain segments to CS, which is conducive to the absorption of starch granules and swelling, and promotes the leaching of amylose starch. While TP significantly reduced the leaching of straight-chain starch from the CS/TP system with the elevated addition of TP (*p* < 0.05), the difference was not significant when the addition amount exceeded 0.6% (*p* > 0.05). This was due to the increase in viscosity with the addition of viscous polysaccharides such as TP, which might hinder the settling of swollen pellets during centrifugation, leading to a decrease in LA value [39], as well as enhanced intermolecular interactions between CS (mainly leached amylose) and TP through hydrogen bonding, reducing the free amylose content in the mixture [40]. Previous studies have shown similar phenomena in rice starch/hydrocolloid (xanthan gum or hydroxypropyl methylcellulose) mixtures [41].

### 3.3. Solubility and Swelling Power

**Figure 4:**
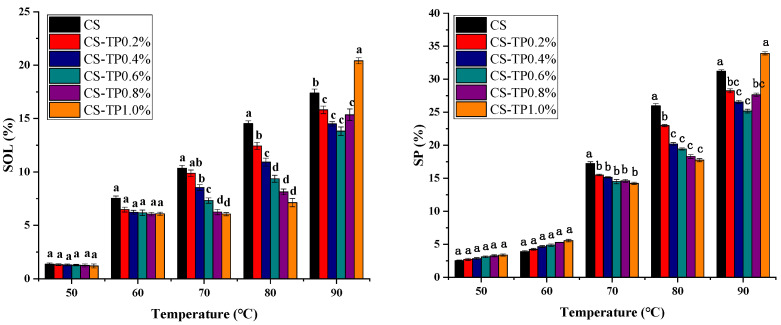
Solubility and swelling of CS/TP composite systems. a–d: Different lowercase letters indicate significant difference (*p* < 0.05).

The solubility and swelling of CS at different temperatures and different concentrations of TP are shown in Figure 4. The SOL and SP of starch are related to the content of amylose and amylopectin, the ratio and the molecular weight. The swelling and solubility of starch increased with the increase in temperature. The SP and SOL of starch reflected the water-absorbing capacity of starch.

In the range of 50–90 °C, the SOL and SP of CS/TP showed a tendency of decreasing and then increasing with the increase of TP addition. First, TP addition inhibited the free SP of starch granules, which may be due to the strong water absorption ability of TP, which competed with starch granules for obtaining water molecules during the pasting process, while TP wrapped around starch granules and inhibited the leaching of amylose, which in turn inhibited the swelling of starch granules [42]. Especially at 90 °C, the SOL and SP of the CS/TP composite system were significantly reduced (*p* < 0.05) with 0.6% TP addition compared to the control sample of CS, reflecting the fact that TP incorporation promotes the formation of a highly ordered molecular structure of CS complexes and that CS/TP composites at the right amount of concentration (0.6%) have an excellent thermal stability against heat. This result was in agreement with the results of the pasting characteristics. At 90 °C, the solubility and swelling of the CS-TP1.0% complex system was slightly higher than that of CS, probably due to the high viscosity of TP.

### 3.4. Rheological Properties

#### 3.4.1. Static Rheological Properties

**Figure 5:**
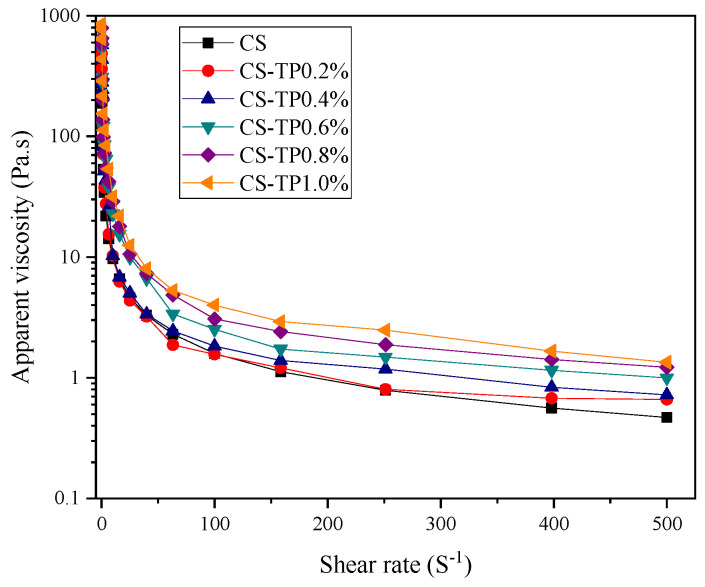
Apparent viscosity of CS/TP composite systems.

The apparent viscosity change curve of the CS/TP complex system is shown in Figure 5. The results show that the apparent viscosity of the CS/TP complex decreases with the increase of shear rate, and the pattern of change is the same for each sample. The apparent viscosity is higher at low shear, and the n value decreases significantly with the increase in shear rate, which belongs to the typical shear-thinning behaviour. At 0.1~10 s^−1^, the apparent viscosity decreases sharply, and the apparent viscosity tends to be stable when the shear rate is up to 100 s^−1^, which is a typical shear-thinning behaviour and presents a non-Newtonian fluid nature. First, the addition of TP caused the CS/TP system to show a gradual increase in the apparent viscosity compared to the single CS, a result similar to the peak viscosity results observed for the pasting process. This suggests that the addition of TP increased the apparent viscosity of CS, which was related to the thickening effect of TP and the hydrogen bonding between TP molecules and straight-chain starch molecules. This phenomenon was also found in the rheological properties of xanthan gum and tapioca starch [43].

In order to further describe the rheological behaviour of the CS/TP system, we fitted the static rheological curves with the power law equation, and the results are shown in Table 2. The coefficients of determination, R^2^, for each sample were all greater than 0.99, indicating that the power law model has good fitting accuracy for the static rheological properties of the CS/TP starch blending system. Among the parameters of the power law model, the fluid indices n are less than 1, indicating that both CS and CS/TP starch systems are pseudoplastic fluids. The value of the consistency coefficient K is related to the amount of TP added. The K value of the composite system increases with the increase of TP, indicating that the thickening of the system is enhanced. This may be related to the intermolecular entanglement or interaction between the hydrophilic gum and starch molecules, which ultimately leads to the increase of K [44]. The trend of the K value is consistent with the effect of peak viscosity of the CS/TP system in RVA experiments.

#### 3.4.2. Dynamic Viscoelasticity

**Figure 6:**
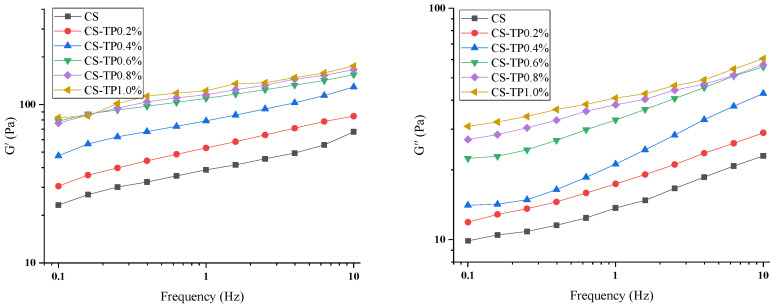
Effect of G′, G″ of composite systems with different additions of TP with CS.

The intermolecular connections and interaction forces are usually described by dynamic rheological measurements, and the corresponding parameters include storage modulus (G′) and loss modulus (G″) to reflect the toughness and rigidity of the gel network, which is closely related to the reconstitution of the pasted starch [32]. The slurries of CS and CS/TP mixtures after the RVA procedure were cooled to 25 °C. Figure 6 shows that the G′ and G″ of all the samples increased with the increase of frequency, and the addition of TP significantly changed the dynamic modulus (G′ and G″) of the starch gels, which suggests that the addition of TP can change the structure of the starch gel and that there is a significant frequency dependence and enhanced interaction between TP and CS effect [45]. It is clear that the G′ values of all samples are always higher than the G″ values throughout the oscillation frequency range, which indicates that the elastic properties dominate the system and all samples show a typical weak gel behaviour [46]. Similar results were observed in studies of MCP–sweet potato starch [45], MCP–tapioca starch paste [47] and barley β-D glucan-wheat starch [48]. In addition, the values of G′ and G″ increased significantly (*p* < 0.05) with the increase of TP addition, suggesting that the viscoelasticity of CS/TP gels was enhanced by the addition of TP. This phenomenon may be attributed to the increase of intermolecular junction regions and entanglement nodes or connection tightness between molecular chains in the hybrid system, which resulted in the formation of a stronger network structure [49]. Meanwhile, our previous studies have shown that TP has good gelling and thickening abilities [50,51]. Therefore, TP can form a hydrated film on the surface of the particles which encapsulates or wraps the particles, thus enhancing the rigidity of the particles and improving the network structure of the gel, which can also explain the higher viscosity of the CS/TP composite gels in the RVA test.

### 3.5. In Vitro Digestive Properties

As can be seen from Table 3, the digestive properties of CS and CS/TP composite systems were characterised by the content of RDS, SDS and RS. However, foods with excessive RDS content or fast digestion rates can lead to various chronic diseases such as diabetes mellitus [27]. Reducing the rate of starch digestion by increasing the content of SD and RS in food is not only beneficial to controlling the blood glucose level of the human body but also improves the feeling of satiety after meals, thus reducing the intake of other foods and achieving weight control. As shown in Table 3, the addition of TP was able to increase the content of SDS + RS in the samples, suggesting that TP has the potential to regulate the starch digestive characteristics, but when the amount of TP added was more than 0.6%, the increase in the content of SDS + RS was not significant (*p* > 0.5). With the increase of TP addition, the RDS content showed a decreasing trend, while the SDS and RS content increased all the time. This might be due to the decrease in the relative content of digestible starch caused by the addition of TP and thus the increase of SDS and RS content. When the amount of TP was 0.6%, the content of RS was the highest (8.83%), which indicated that the effect of TP on the RS content of CS was complex. The effect of TP on the digestibility of the CS/TP blended system was the result of the combination of several factors. Firstly, starch digestion is mainly preceded by the processes of pasting and short-term regeneration, in which TP interacts with straight-chain starch and branched-chain starch to form larger aggregates and reduce the contact between enzymes and starch, thus slowing down the digestion of starch. Secondly, TP can compete with starch for water molecules and inhibit the swelling of starch granules, resulting in incomplete pasting of some starch granules and thus reducing the starch digestion rate. In addition, when TP is added in larger amounts, it may wrap around the surface of starch granules and reduce the accessibility of digestive enzymes to starch molecules, thus inhibiting starch digestion. In conclusion, the addition of TP decreased the RDS content and increased the SDS and RS content of CS, thus suggesting that TP may reduce the TS digestion rate and help to reduce postprandial blood glucose and insulin levels [52]. These results are similar to the findings of wheat starch blended with MCP [53].

### 3.6. In Vitro Digestion Kinetics

**Figure 7:**
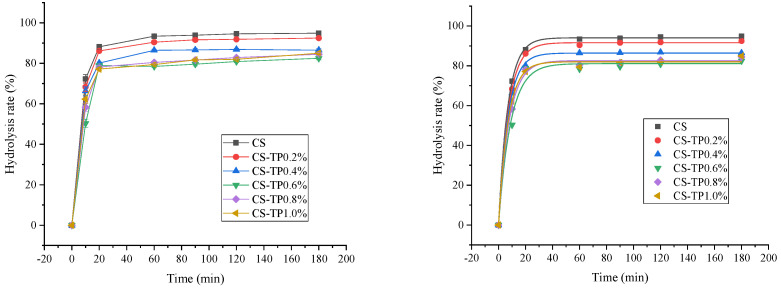
In vitro digestion profiles of mixtures of the CS/TP composite system.

In order to better understand the effect of TP on the in vitro digestion process of CS, we analysed the CS/TP composite system using digestion kinetics and used a first-order equation model to estimate the maximum percentage of starch hydrolyses for digestion (C_∞)_ and the first-order digestion rate coefficient (K) [54]. The digestion profiles of the CS and CS/TP composite systems and their kinetic fitting parameters are shown in Figure 5 and Table 4, respectively. TS showed a faster hydrolysis rate (17.71%) in the first 60 min, which was reduced by the addition of TP. This situation may be caused by viscosity. The first-order equation model for starch hydrolysis was well fitted with R^2^ values ranging from 0.9803 to 0.9916 in 0–180 min (Table 4). K values show the change in digestibility during digestion. As the TP content increased to 0.6%, the K value decreased from 0.1003 min^−1^ to 0.0706 min^−1^. This indicates that the addition of TP decreased the digestibility of CS. The K value decreased and then increased, which may be attributed to the fact that the addition of TP reduces the exudation of straight-chain starch during starch pasting and interacts with the exuded straight-chain starch, which slows down the hydrolysis by the enzyme. However, from the digestion curve (Figure 7), the digestion was initially slower due to the presence of TP. The C∞ value decreased from 94.07 g/100 g for the CS sample to 78.99 g/100 g for the 0.6% CS/TP sample (Table 4), suggesting that the 0.6% addition of TP reduced not only the digestibility but also the amount of CS digested. The results of the study had a similar effect regarding the retardation of starch digestion by Nigella sativa polysaccharides, pectin, etc. [55]. In addition, we found that the inhibitory effect of TP on CS digestion may originate from the fact that, on the one hand, TP forms a physical barrier layer around the starch molecules, preventing the enzyme from contacting the starch molecules [56]. On the other hand, the ability of TP to protect the CS crystal structure promotes the formation of a stronger ordered structure and a more compact double-helix structure, which limits the accessibility of starch to digestive enzymes to a certain extent and makes the composite system the most resistant to enzyme digestion. This is in agreement with the crystallinity determined by X-RD and the FT-IR ordering results. The increase in crystallinity and the degree of starch ordering directly affected the ease of access of water molecules to the starch granules and the contact of amylase.

### 3.7. Particle Size Distribution

The results of the particle size of the CS/TP compound system after adding different contents of TP are shown in Table 5. From the table, it can be seen that with the introduction of TP, the particle size of the compound system decreased, which may be due to the fact that on the one hand, TP is easily soluble in water, and the more the amount of TP is added, the smaller the concentration of starch is in relation to the starch, resulting in the reduction of the particle size of the compound system. On the other hand, TP may be coated or adhered around the starch granules, inhibiting the swelling of the starch granules, which in turn leads to the reduction of the granule size in the compound system. After continuing to increase the concentration of TP, the particle size and system in the composite system increased slightly. It is noteworthy that the swelling of starch granules was more dominant throughout the pasting process, as the mean volume diameter was affected by the full range of granule swelling and rupture. These findings are consistent with the results of the RVA and rheological tests described in the article.

### 3.8. Thermal Properties

**Figure 8:**
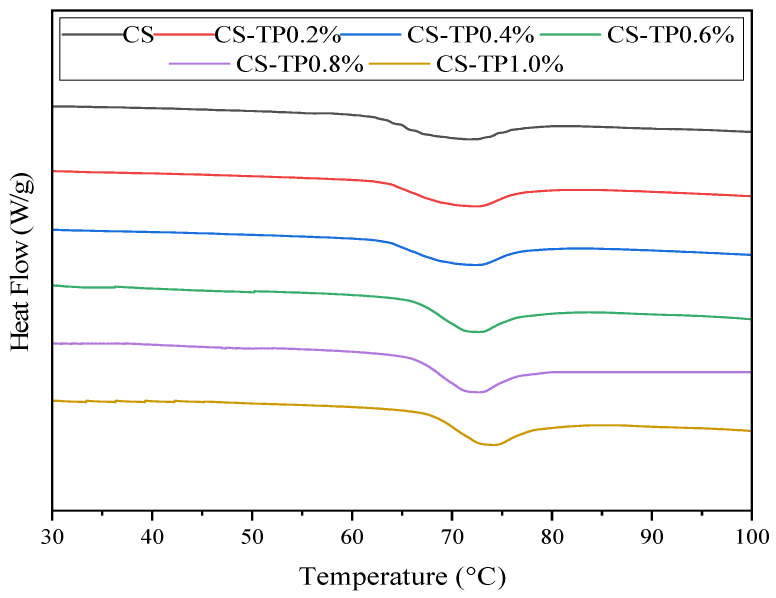
Thermodynamic characteristic curves of CS/TP composite system.

Figure 8 shows the heat flow curves of the samples when they were heated for the first time, from which it can be clearly seen that with the addition of TP, the pasting temperatures of starch, T_O_, T_P_ and T_C_ were increased to different degrees, which further illustrates that the addition of TP made the pasting of CS increasingly difficult; in fact, the pasting of the starch in the system was not sufficient but only partial. In addition, TP is very hydrophilic, and a higher addition will change the nature of water molecules in the system, especially those close to the starch granules, and restrict the motility of these water molecules, thus inhibiting the water absorption, swelling and pasting of the starch granules. The increase of the pasting temperature T_O_ and T_P_ of starch further indicated that after the addition of TP, a structurally stable network structure was formed in the composite system, which played a protective role for the CS crystal structure and reduced the mobility of the starch chain. It is consistent with the previous results of the pasting characteristics study.

TP significantly reduced the ΔH value of the composites (*p* < 0.05) in Table 6. This phenomenon is in agreement with the results of the enthalpy change of the pasting of potato starch by TP and the enthalpy change of the pasting of potato starch by KGM [57,58]. During the heating and pasting process, TP has strong water absorption and will compete with starch molecules for water, thus limiting the contact between TP and water molecules. Thus, the starch cannot be completely decomposed or the heat absorbed, leading to incomplete pasting and a decrease in ΔH. Therefore, the main reason for the decrease in the ΔH value of the composites could be that TP makes the pasting of CS incomplete because of the reduced availability of water [59]. In addition, the interaction between TP and straight-chain starch alters the structure of the starch paste, leading to defects in the starch crystal region [60]. In conclusion, the addition of TP increased the pasting temperature and decreased the ΔH value of CS. This result can be explained as a result of incomplete starch pasting induced by TP.

### 3.9. Crystalline Structure

**Figure 9:**
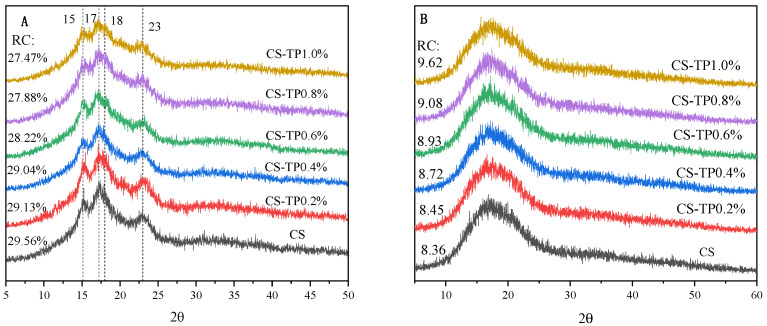
XRD of CS/TP composite systems. (**A**) unpasteurised, (**B**) pasteurised.

XRD is an effective method to characterise the crystal structure of starch, and the position of the diffraction angle 2θ can characterise the crystal type of starch. Different types of starch have specific diffraction peaks, which can be classified into A-type, B-type, C-type and V-type according to the ray diffraction pattern. As shown in Figure 9, CS has characteristic peaks at 15°, 17°, 18° and 23°, which is a typical A-type crystal structure [61]. Compared to unpasteurised CS, when TP was added, all samples showed strong diffraction peaks at 15°, 17°, 18° and 23°, indicating that the addition of TP did not change the A-type crystalline type of CS (Figure 9A). Moreover, the addition of TP did not affect the diffraction peaks located at 2θ in CS, indicating that TP does not interfere with the natural complex formed by starch and lipids. The addition of TP slightly decreased the crystallinity (RC) of CS. This was mainly due to the addition of TP (amorphous structure) (Figure 9A). Comparing the XRD of CS and CS/TP after pasting (Figure 9B), it is easy to find that heat pasting caused a change not only in the crystalline shape of starch but also in the width of the diffraction peaks, which indicates that pasting causes serious damage to the granular morphology of starch, damages the crystalline region of starch and inhibits the reordering of straight-chained starch. The crystallinity of unpasteurised CS was as high as 29.56, while pasting caused a significant decrease in its relative crystallinity to 8.36 (Figure 9B). With the increase of TP addition, the relative crystallinity of the CS/TP blended system increased, and it was more obvious at 1.0% addition, which was higher than that of CS by 15.7%, indicating that TP interspersed in the crevices of starch during the pasteurisation stage could play a role in protecting the crystalline structure of CS from being disrupted by the ordered structure of CS. This result is consistent with the results of FT-IR. The strengthening of the crystalline structure helps to hinder the action of digestive enzymes, which also plays a certain role in the autodigestive action of starch.

### 3.10. Short-Range Ordered Structures

**Figure 10:**
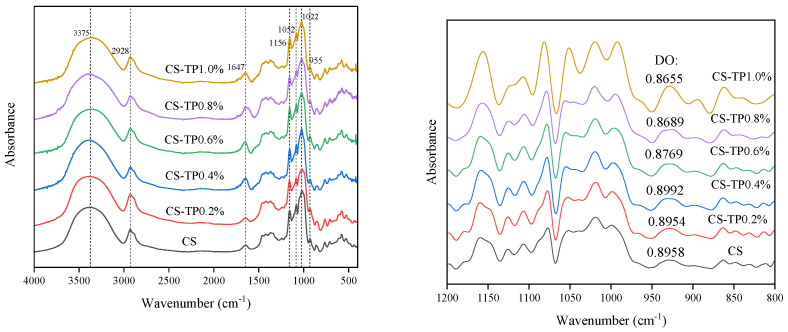
FT-IR and deconvolution spectra of CS/TP composite system.

The FT-IR of CS and CS/TP composite systems in the range of 400–4000 cm^−1^ wavelength are observed in Figure 10, and it is found that the shape and position of the infrared absorption peaks of the CS/TP composite system are essentially similar to those of the original starch CS. There is no appearance of new absorption peaks or disappearance of absorption peaks, which suggests that the CS/TP composite system has not formed any new groups, i.e., there is no covalent bonding involved. Since both CS and TP are polysaccharides, containing a large number of hydroxyl groups, there are glycosidic bonds in the structure, which make a large number of hydrogen bonds formed in the intermolecular. The CS/TP composite system formed a broad absorption peak in the range of 3000–3700 cm^−1^, which was due to the intermolecular hydroxyl O-H stretching vibration, and it was a typical characteristic peak of intermolecular hydroxyl bonding of polymers; and with the increase of TP addition, the characteristic peak gradually moved to the lower wave number and the intensity of the peak became bigger, which indicated that there existed the interaction of non-covalent bonding, such as hydrogen bonding, between starch and TP. The absorption peak near 2920 cm^−1^ is the C-H group stretching vibration. In addition, the bending vibration of -OH water was near 1647 cm^−1^, which is the part of bound water in starch [62]. Previous studies have also reported that TP can bind to starch through non-covalent bonds such as hydrogen bonding, electrostatic forces or hydrophobicity, rather than through covalent binding [59,63]. Due to the large number of hydroxyl groups contained in TP itself, it may bind to the hydroxyl groups in starch through hydrogen bonding, thus covering the surface of CS granules, inhibiting the leakage of straight-chain starch and the swelling of starch granules, and decreasing the degree of pasting of CS.

The IR absorption peaks in the range of 800–1200 cm^−1^ can be used to characterise the short-range ordering of starch. 1045/1022 cm^−1^ (DO) was used to determine the short-range ordering of starch molecules. From Figure 8, it can be seen that the peak intensities at 1045 cm^−1^, 1022 cm^−1^ and 995 cm^−1^ gradually increased with the increase of TP addition; especially the peak intensities at 1022 and 995 cm^−1^ increased more significantly. This further suggests that TP has a positive effect on maintaining the crystalline region and amorphous going of starch molecules. It indicates that the ratio of DO of the CS/TP composite system shows a gradual increase; i.e., the degree of short-range ordering of the CS increases, indicating that the addition of TP has a contributing effect on the maintenance of the degree of short-range ordering of CS. This is consistent with the crystallinity results determined by X-ray diffraction. We speculate that the addition of TP played a protective effect on the crystal structure of CS and that TP promoted the formation of a stronger ordered structure and a more compact double-helix structure of CS after the reorientation of starch molecules, which limited the accessibility of digestive enzymes to starch to a certain extent and rendered the composite system the most resistant to digestion by enzymes. In addition, the increase in the DO value of the CS/TP composite system with 0.8–1.0% additions was not significant (*p* > 0.05). The degree of starch ordering has always been considered to be significantly positively correlated with starch digestibility, and the degree of starch ordering directly affects the ease of water molecules entering starch granules and contacting amylase. In conclusion, the addition of TP can hinder the entanglement of starch chains and the formation of hydrogen bonds, which can lead to the decrease of gel strength and digestibility properties of CS.

### 3.11. Microstructural Morphology

**Figure 11:**
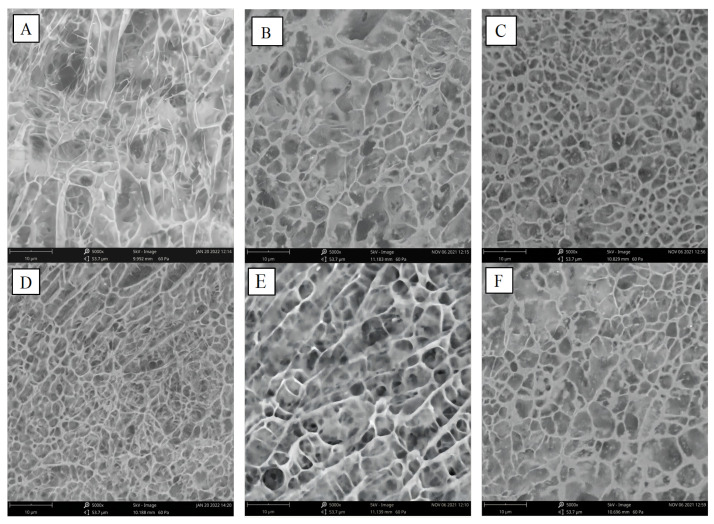
Cryo-electron microscopy (Cryo-SEM) images of CS/TP composite systems. (**A**) CS; (**B**) CS-TP0.2%; (**C**) CS-TP0.4%; (**D**) CS-TP0.6%; (**E**) CS-TP0.8%; (**F**) CS-TP1.0%.

Figure 11 depicts the microstructure of CS and CS/TP gels. All samples showed a “honeycomb” structure. The gel structure formed by CS alone was rough, fragile, disordered and weakly matrixed, with uneven, irregular and discontinuous mesh arrangement, loose structure, and a large number of holes and depressions (Figure 11A), indicating that the CS gels were more susceptible to the erosion by digestive enzymes. The addition of TP significantly changed the microscopic network structure of starch gels (Figure 11B–F). All composite gels showed a continuous and relatively ordered lamellar network structure with parallel strands and thicker pore walls (Figure 11B–F). With the increase of TP addition, the network structure of the composites was gradually stabilised, and the number of pores was reduced, forming a uniform and continuous structure with special features of interactive entanglement and connecting bridge matrix (Figure 11B–F). With the increase in TP concentration, the composites showed a more compact and regular microstructure with smaller voids. This may be due to the intertwining effect of TP with the leached starch, which makes the pore walls thicker and more stable, and thus less prone to decomposition in the presence of digestive enzymes [58,64,65]. In addition, polysaccharides compete with starch for water, so less water is leached into the starch granules, resulting in a smaller number of pores. Yuris [66] and Kaur [67] also came up with similar results by studying the microstructure of polysaccharide/starch composites, respectively. Strikingly, a denser and stronger three-dimensional network structure with tight overlapping and stacking was observed in CS-TP0.6% gels (Figure 11D). At additions greater than 0.8%, the structure tended to be associated with disorder, which was attributed to the increase in TP addition and the decrease in the relative starch content, leading to either the evacuation of the network structure or the gel structure of the surface-attached polysaccharides themselves being revealed. These images suggest that the enhanced synergy between TP and CS effectively contributes to the formation of a more stable microstructure. This result also supports the experimental results of FT-IR spectroscopy that TP can help CS/TP gels to form more ordered structures. As long as the appropriate amount of TP is added, it can help CS/TP gels to form better microstructures, which in turn can play a certain positive role in antidigestion.

### 3.12. Correlation Analysis

**Figure 12:**
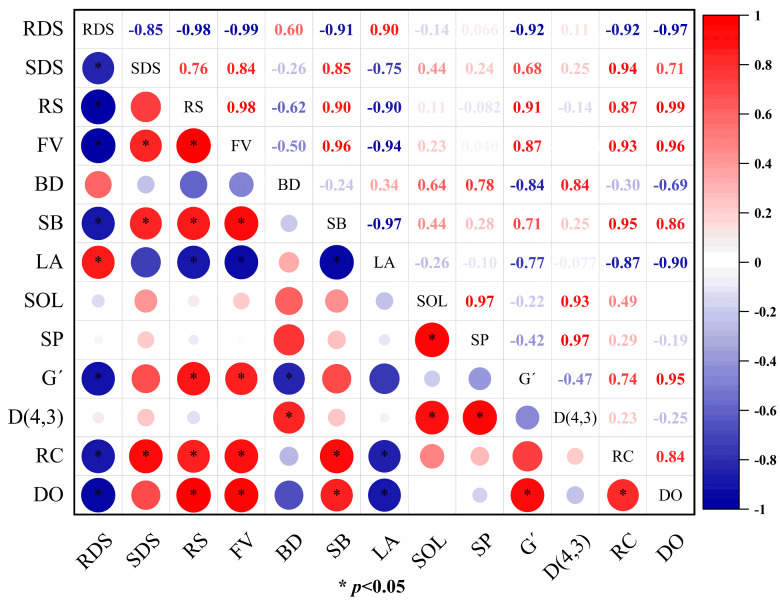
Pearson’s correlation between in vitro starch digestibility and other key parameters for CS/TP composite systems.

In order to better understand the relationship between the solubility, pasting properties, rheological properties, crystallinity, order and in vitro digestibility of the CS/TP composite system, we performed Pearson’s correlation analysis, and the results are shown in Figure 12. We found that the in vitro digestibility properties of the CS/TP composite system in terms of RDS showed a significant positive correlation with LA (r = 0.90, *p* < 0.05) and a positive correlation with BD (r = 0.60); they significantly negatively correlated with FV (r = −0.99, *p* < 0.05), SB (r = −0.91, *p* < 0.05), G′ (r = −0.92, *p* < 0.05), RC (r = −0.92, *p* < 0.05) and DO (r = −0.97, *p* < 0.05). While SDS was significantly positively correlated with FV (r = 0.84, *p* < 0.05), SB (r = 0.85, *p* < 0.05) and RC (r = 0.94, *p* < 0.05), it was only negatively correlated with LA (r = −0.75). Notably, RS content was significantly positively correlated with FV (r = 0.98, *p* < 0.05), SB (r = 0.90, *p* < 0.05), G′ (r = 0.91, *p* < 0.05), RC (r = 0.87, *p* < 0.05) and DO (r = 0.99, *p* < 0.05) and strongly inversely correlated with LA (r = −0.90, *p* < 0.05). We also found that D(4,3) was positively correlated with SP (r = 0.97, *p* < 0.05), SOL (r = 0.93, *p* < 0.05) and SB (r = 0.84, *p* < 0.05), respectively. The above data confirm that the addition of TP improves the viscoelasticity, crystallinity and short-range ordering of the composite gel, which is conducive to reducing starch digestibility. It is well known that postprandial glucose regulation by slowing down the rate of starch digestion to prevent diseases such as obesity and diabetes is also an effective dietary intervention management method [68].

## 4. Conclusions

Starch is an important component of the human diet and the main source of energy. The influence of starch intake on blood glucose and lipids has become the focus of current research. The physicochemical, structural and digestive properties of starch can be significantly influenced by other components of food, such as polysaccharides, proteins, lipids, etc. In addition, relevant studies have shown that other food components such as polysaccharides can affect the gelatinisation, retrogenesis and viscoelasticity of starch through intermolecular or intramolecular forces (hydrogen bonding, electrostatic interaction and steric hindrance), and then affect the changes in the crystal structure and double helix structure of starch, which are key factors affecting the digestibility of starch products. In this paper, the effects of TP on gelatinisation properties, particle expansion, particle size distribution, static rheology, dynamic viscoelasticity, crystalline and ordered structure, and in vitro digestive properties of CS were discussed. The conclusions are as follows:

The results showed that the addition of TP increased the gelatinisation viscosity and temperature of CS, and inhibited the gelatinisation of CS. The increase of TP content (0.4–1.0%) inhibited the leaching of amylose, and the leaching amount of amylose with 1.0% TP decreased by 38%. TP can increase the T_O_, T_P_ and T_C_ of CS, and significantly reduce their ΔH (*p* < 0.05). The addition of TP significantly changed the dynamic modulus of starch gel (G′, G″) (*p* < 0.05) and enhanced the viscoelasticity of the system in a dose-dependent manner. TP mainly interacted with CS through non-covalent bonds such as hydrogen bonds. TP at 0.2–0.6% significantly enhances the order degree (OD) (*p* < 0.05), crystal structure and stability of CS/TP complex network structure, and enhances the surface structure density of the system. Meanwhile, an in vitro digestibility study showed that TP effectively reduced the content of RDS in the CS/TP complex, but significantly increased the content of SDS and RS (*p* < 0.05). When the content of TP was 0.6%, the digestibility of starch was the smallest, the content of RDS was decreased by 12.6%, the content of SDS and RS was increased by 80.5% and the maximum percentage of starch hydrolysis (C_∞_) was 78.99%. The Pearson correlation showed that the SDS and RS content correlated well with G′, FV, RC and DO. Therefore, TP could play a role in reducing the digestibility of CS by increasing the viscoelasticity of the system, inhibiting starch pasting, enhancing the relative crystallinity and short-range ordering, maintaining the integrity of the starch granule and crystalline structure, and enhancing the gel network structure of the system. This study presents the modulating effect of TP on the in vitro digestive properties of CS from multiple perspectives, such as pasting, rheology and multiscale structure, and thus provides new insights into the application of CS/TP systems.

## Figures and Tables

**Table 1:** Pasting performance parameters of CS/TP composite systems.

Samples	PV/mPa·s	TV/mPa·s	BD/mPa·s	FV/mPa·s	SB mPa·s	PT/°C
CS	7276.67 ± 29.09 ^e^	3465.00 ± 13.53 ^e^	3811.67 ± 25.42 ^a^	4380.67 ± 15.14 ^d^	915.61 ± 28.29 ^d^	72.12 ± 0.40 ^c^
CS-TP0.2%	7384.26 ± 21.05 ^e^	3788.82 ± 19.20 ^d^	3595.44 ± 12.03 ^c^	4630.33 ± 12.38 ^d^	841.51 ± 11.36 ^e^	73.66 ± 0.22 ^b^
CS-TP0.4%	7637.83 ± 18.99 ^d^	4069.11 ± 11.13 ^c^	3568.72 ± 16.31 ^b^	5020.33 ± 18.21 ^d^	951.22 ± 17.42 ^d^	74.08 ± 0.13 ^b^
CS-TP0.6%	8226.33 ± 36.06 ^c^	4830.00 ± 12.37 ^b^	3396.33 ± 14.16 ^c^	5902.34 ± 17.52 ^c^	1072.34 ± 14.79 ^c^	76.15 ± 0.04 ^a^
CS-TP0.8%	8534.12 ± 18.59 ^b^	5013.25 ± 16.18 ^a^	3520.87 ± 13.08 ^b^	6187.67 ± 16.81 ^b^	1174.42 ± 14.25 ^b^	76.52 ± 0.09 ^a^
CS-TP1.0%	8774.37 ± 19.87 ^a^	5128.23 ± 17.32 ^a^	3646.14 ± 14.62 ^b^	6391.13 ± 13.22 ^a^	1262.90 ± 16.17 ^a^	76.93 ± 0.15 ^a^

Data are presented as mean ± standard deviation in triplicate. Different superscript letters in the same column indicate significant differences (*p* < 0.05).

**Table 2:** Parameter values of static rheological power law equations for composite systems with different additions of TP with CS.

Samples	K (Pa·s)^n^	*n*	R^2^
CS	47.478 ± 1.05 ^a^	0.158 ± 0.006 ^a^	0.998
CS-TP0.2%	63.690 ± 2.88 ^b^	0.132 ± 0.005 ^b^	0.990
CS-TP0.4%	97.919 ± 5.56 ^c^	0.124 ± 0.003 ^c^	0.991
CS-TP0.6%	112.774 ± 4.76 ^d^	0.112 ± 0.002 ^c^	0.990
CS-TP0.8%	139.904 ± 3.55 ^e^	0.105 ± 0.001 ^c^	0.994
CS-TP1.0%	156.369 ± 3.89 ^f^	0.113 ± 0.002 ^c^	0.996

Data are presented as mean ± standard deviation in triplicate. Different superscript letters in the same column indicate significant differences (*p* < 0.05).

**Table 3:** In vitro digestion parameters of the CS/TP composite system.

Samples	RDS/%	SDS/%	RS/%	SDS + RS/%
CS	88.33 ± 0.23 ^a^	8.91 ± 0.31 ^c^	2.76 ± 0.14 ^e^	11.67 + 0.22 ^d^
CS-TP0.2%	86.00 ± 0.44 ^b^	10.15 ± 0.56 ^bc^	3.85 ± 0.18 ^d^	14.00 + 0.14 ^c^
CS-TP0.4%	83.72 ± 0.52 ^c^	11.88 ± 0.33 ^b^	4.40 ± 0.21 ^c^	16.28 + 0.16 ^b^
CS-TP0.6%	78.93 ± 0.24 ^d^	12.24 ± 0.35 ^b^	8.83 ± 0.43 ^a^	21.07 + 0.25 ^a^
CS-TP0.8%	78.09 ± 0.25 ^cd^	13.79 ± 0.57 ^a^	8.12 ± 0.48 ^a^	21.91 + 0.33 ^a^
CS-TP1.0%	79.57 ± 0.76 ^c^	13.26 ± 0.42 ^a^	7.17 ± 0.19 ^b^	20.43 + 0.27 ^a^

Data are presented as mean ± standard deviation in triplicate. Different superscript letters in the same column indicate significant differences (*p* < 0.05).

**Table 4:** Digestion kinetic parameters of the composite system with different amounts of added TP and CS.

Samples	C_∞_ (%)	K (min^−1^)	R^2^
CS	94.07 ± 0.30 ^e^	0.1405 ± 0.0003 ^b^	0.993
CS-TP0.2%	88.41 ± 2.85 ^d^	0.1003 ± 0.016 ^b^	0.989
CS-TP0.4%	83.85 ± 0.33 ^c^	0.0941 ± 0.017 ^b^	0.980
CS-TP0.6%	78.99 ± 1.48 ^a^	0.0706 ± 0.006 ^a^	0.992
CS-TP0.8%	80.18 ± 2.23 ^b^	0.0782 ± 0.012 ^a^	0.981
CS-TP1.0%	81.65 ± 2.28 ^b^	0.0794 ± 0.013 ^b^	0.992

Data are presented as mean ± standard deviation in triplicate. Different superscript letters in the same column indicate significant differences (*p* < 0.05).

**Table 5:** Particle size distribution of CS/TP composite systems.

Samples	D(3,2)	D(4,3)	D10	D50	D90
CS	8.62 ± 0.35 ^a^	15.4 ± 0.19 ^a^	5.12 ± 0.08 ^a^	9.14 ± 0.16 ^a^	19.5 ± 0.32 ^a^
CS-TP0.2%	7.11 ± 0.22 ^b^	14.3 ± 0.21 ^b^	4.83 ± 0.11 ^b^	8.77 ± 0.13 ^a^	18.6 ± 0.25 ^a^
CS-TP0.4%	6.40 ± 0.26 ^c^	13.8 ± 0.17 ^b^	4.36 ± 0.09 ^b^	7.28 ± 0.23 ^b^	17.4 ± 0.36 ^b^
CS-TP0.6%	5.32 ± 0.21 ^d^	12.9 ± 0.43 ^c^	3.15 ± 0.14 ^c^	4.83 ± 0.11 ^c^	15.6 ± 0.33 ^c^
CS-TP0.8%	5.64 ± 0.18 ^d^	13.3 ± 0.12 ^bc^	4.08 ± 0.06 ^b^	5.27 ± 0.15 ^d^	16.9 ± 0.18 ^b^
CS-TP1.0%	7.23 ± 0.32 ^b^	15.8 ± 0.15 ^a^	5.36 ± 0.07 ^a^	6.60 ± 0.18 ^c^	17.4 ± 0.21 ^b^

Data are presented as mean ± standard deviation in triplicate. Different superscript letters in the same column indicate significant differences (*p* < 0.05).

**Table 6:** Thermodynamic property parameters of CS/TP composite system.

Samples	T_O_/°C	T_P_/°C	T_C_/°C	∆H (J/g)
CS	63.56 ± 0.11 ^e^	71.15 ± 0.02 ^e^	76.88 ± 0.04 ^c^	12.84 ± 0.23 ^e^
CS-TP0.2%	64.31 ± 0.12 ^d^	71.53 ± 0.05 ^e^	77.65 ± 0.01 ^b^	11.48 ± 0.11 ^d^
CS-TP0.4%	65.48 ± 0.05 ^c^	72.95 ± 0.03 ^d^	78.03 ± 0.02 ^a^	10.07 ± 0.13 ^c^
CS-TP0.6%	67.11 ± 0.08 ^b^	73.69 ± 0.08 ^c^	78.28 ± 0.08 ^a^	9.34 ± 0.09 ^b^
CS-TP0.8%	68.16 ± 0.09 ^a^	74.82 ± 0.04 ^b^	78.56 ± 0.06 ^a^	8.23 ± 0.06 ^a^
CS-TP1.0%	68.13 ± 0.04 ^a^	75.32 ± 0.06 ^a^	78.93 ± 0.03 ^a^	8.04 ± 0.07 ^a^

Data are presented as mean ± standard deviation in triplicate. Different superscript letters in the same column indicate significant differences (*p* < 0.05).

## Data Availability

The original contributions presented in the study are included in the article; further inquiries can be directed to the corresponding author.
